# Escape from the cryptic species trap: lichen evolution on both sides of a cyanobacterial acquisition event

**DOI:** 10.1111/mec.13636

**Published:** 2016-05-11

**Authors:** Kevin Schneider, Philipp Resl, Toby Spribille

**Affiliations:** ^1^ Institute of Plant SciencesNAWI GrazUniversity of GrazHolteigasse 6A‐8010GrazAustria; ^2^ Institute of ZoologyUniversity of GrazUniversitätsplatz 2A‐8010GrazAustria; ^3^ Division of Biological SciencesUniversity of Montana32 Campus DriveMissoulaMT59812USA

**Keywords:** apothecia, fungi, nutrient flows, sexual reproduction, speciation, symbiosis

## Abstract

Large, architecturally complex lichen symbioses arose only a few times in evolution, increasing thallus size by orders of magnitude over those from which they evolved. The innovations that enabled symbiotic assemblages to acquire and maintain large sizes are unknown. We mapped morphometric data against an eight‐locus fungal phylogeny across one of the best‐sampled thallus size transition events, the origins of the *Placopsis* lichen symbiosis, and used a phylogenetic comparative framework to explore the role of nitrogen‐fixing cyanobacteria in size differences. Thallus thickness increased by >150% and fruiting body core volume increased ninefold on average after acquisition of cyanobacteria. Volume of cyanobacteria‐containing structures (cephalodia), once acquired, correlates with thallus thickness in both phylogenetic generalized least squares and phylogenetic generalized linear mixed‐effects analyses. Our results suggest that the availability of nitrogen is an important factor in the formation of large thalli. Cyanobacterial symbiosis appears to have enabled lichens to overcome size constraints in oligotrophic environments such as acidic, rain‐washed rock surfaces. In the case of the *Placopsis* fungal symbiont, this has led to an adaptive radiation of more than 60 recognized species from related crustose members of the genus *Trapelia*. Our data suggest that precyanobacterial symbiotic lineages were constrained to forming a narrow range of phenotypes, so‐called cryptic species, leading systematists until now to recognize only six of the 13 species clusters we identified in *Trapelia*.

## Abstract 2

See also the Perspective by Scheidegger

## Introduction

The oligotrophic nature of rock surfaces, where elements such as carbon and nitrogen, essential for every living being, are scarce, poses major challenges to the sessile organisms growing on them. For heterotrophic organisms such as fungi, there are basically four possibilities to overcome this problem. The first one involves living off of windborne detritus or matter distributed by nonsessile organisms. The second way is the weathering of the rock surface to release, besides other nutrients, carbon from carbonate rock, such as limestone or dolomite, or nitrogen from ammonium‐bearing sedimentary rock or bedrock (Chen *et al*. 2000; Holloway & Dahlgren 2002). The third possibility, common throughout the fungal kingdom and present in many biofilms, is to parasitize on the living or degrade the dead tissues of other organisms (Hawksworth 1982; Lawrey & Diederich 2003; Gorbushina 2007). Lastly, the fourth option is to form symbioses with autotrophic organisms that fix atmospheric carbon and, in some cases, even nitrogen. Only this fourth possibility allows an organism to overcome the dependency on organic remnants of others and become pioneers on previously uninhabited surfaces. It is the fourth strategy that has propelled the lichen symbiosis to one of the most successful eukaryotic life forms on rocks. Lichens have radiated onto nearly every terrestrial rock surface from the Antarctic dry valleys (Friedmann *et al*. 1980) to 7400 m on Mt. Everest (Hertel 1977; Hafellner 1987).

A recurring feature of many rock‐dwelling lichens is the narrow range of phenotypes they develop. The thallus, far from being well developed and striking as in the popular image of a lichen, is in rock‐dwelling species often little more than a biofilm measuring tens of microns thick, typically ringing the most conspicuous feature, the fruiting body or apothecium. Only a handful of basic apothecial growth types have been described and this, combined with a finite number of basic ascospore schemes, led early researchers to assume that rock‐dwelling lichens arose from only a handful of fungal lineages. In the most extreme case, more than 1300 species, including non‐rock‐inhabiting species, were placed in a single genus, *Lecidea*, only to be subsequently found to represent dozens of different convergent lineages (Hedlund 1892; Hertel 1977; Coppins 1983; Printzen 1995; Bendiksby *et al*. 2015). The most recent extension of this trend has been in the discovery, using molecular tools, that many long‐recognized species names are themselves merely fronts for even more genetically distinct but phenotypically virtually indistinguishable species (Ruprecht *et al*. 2010; Orange 2014; Westberg *et al*. 2015), so‐called ‘cryptic species’.

**Figure 1:**
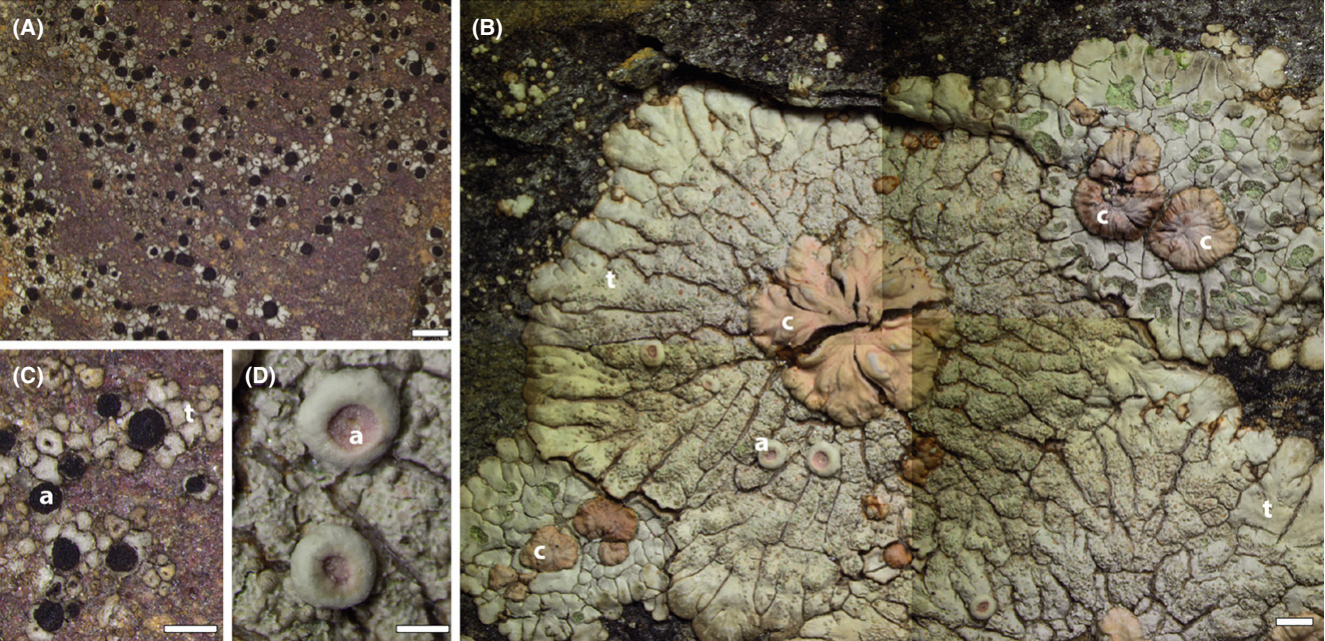
Typical lichen thalli of the *Trapelia*–*Placopsis* clade before (A, C) and after (B, D) the cyanobacterial acquisition event, shown at the same magnification (scale bar for A and B = 1 mm). A. *Trapelia glebulosa*, Austrian Alps; B. *Placopsis cribellans* (centre, large thallus) flanked on top right and bottom left by *Placopsis fusciduloides*, Mitkof Island, Alaska (composite image). C./D. Fruiting structures (apothecia) before (C) and after (D) the cyanobacterial acquisition event at the same magnification (scale bar = 0.5 mm), from the same specimens as (A) and (B). a: apothecia; c: cephalodia; t: thallus.

The discovery and surprising commonness of cryptic species has been a dominant theme in literature on lichen‐forming fungi over the last decade (Crespo & Pérez‐Ortega 2009; Crespo & Lumbsch 2010; Leavitt *et al*. 2011; Singh *et al*. 2015). Few explanations have been proffered as to what mechanisms give rise to cryptic speciation in the absence of visible phenotypes on which natural selection could act. It was against this background that we were intrigued by our recent observation that a large radiation of phenotypically well‐marked species in the cosmopolitan genus *Placopsis* appears to have arisen paraphyletically within a cluster of almost indistinguishable but deeply diverged species in the genus *Trapelia* (Resl *et al*. 2015). *Placopsis* encompasses 60‐plus species with distinct, radiating lobes that form a characteristic ‘bull's eye’ pattern on rocks in maritime climates (Fig. 1), and some can achieve diameters of half a metre (Galloway 2013). It has been recognized as a distinct genus for 155 years (Nylander 1861). *Trapelia* species, by contrast, form nearly featureless thalli only millimetres across (Fig. 1) and were formerly lumped into the above‐mentioned megagenus *Lecidea*; the genus was not widely recognized until the 1970s. In terms of substrate ecology, however, the two are similar: both *Trapelia* and *Placopsis* are early successional pioneers in habitats where little else has colonized a rock surface before. Most species of *Trapelia* and *Placopsis* are obligately rock dwelling, having apparently lost the ability to colonize other substrates (P. Resl and T. Spribille, unpublished).

The preliminary data of Resl *et al*. (2015) seemed to suggest that the two long‐held genera *Trapelia* and *Placopsis* are essentially a monophyletic group, with the only difference in the constitution of their symbiosis: *Placopsis* develops specially modified structures called cephalodia that completely enclose colonies of cyanobacteria as secondary symbionts. Cyanobacterial symbionts have been shown to be capable of both carbon and nitrogen fixation (Hitch & Stewart 1973; Fay 1981; Haselkorn 1986; Rai 1990). Nitrogen fixation is beneficial not only to the cyanobiont but also to both the fungal and algal partners. Raggio *et al*. (2012), for instance, suggested a role of increased nitrogen content in lichens, as enabled by cyanobacterial nitrogen fixation, in increasing the photosynthetic rate. The presence and abundance of cyanobacteria could conceivably be a mechanism to allow *Trapelia,* in the form of *Placopsis*, an ‘escape’ from phenotypic constraint. At the same time, it would suggest that resource limitation may be a factor that, for the rest of *Trapelia*, has impeded the exploration of body plan space over evolutionary time.

The close relationship of *Placopsis* to *Trapelia* has been known for some time (Choisy 1929; Hertel & Leuckert 1969; Hertel 1970; Schmitt *et al*. 2003), but the suggestion of paraphyly (Resl *et al*. 2015) was new and required more rigorous testing. In particular, taxon sampling and the number of available gene loci to date have limited the ability to make greater inferences, and many enigmatic taxa (e.g. *Placopsis roseonigra*, Brodo 1995) had not been sampled. Furthermore, in the absence of a statistically robust morphometric study, the apparent thallus size increase could be observer bias caused by the more pronounced contiguity of thalli in *Placopsis*. To investigate the potential selective advantage encompassed by the acquisition of cyanobacterial symbionts, we attempted (i) to acquire a taxon sampling considerably larger than that of previous studies to rule out the possibility that *Trapelia*–*Placopsis* paraphyly was not merely a sampling artefact, (ii) to elucidate the distribution of thallus and hymenial volumes as proxies for lichen biomass over a *Trapelia*–*Placopsis* phylogeny and (iii) to analyse the correlation of thallus and cephalodial volume as well as hymenial and cephalodial volume, where the volume of cephalodia serves as a proxy for cyanobacterial mass and thus the potential for nitrogen uptake. Hereby, we wanted to clarify the potential importance of nitrogen input through symbionts in the apparent increase in lichen biomass in the discussed evolutionary transition from *Trapelia* to *Placopsis* in the face of low nitrogen availability on nutrient‐poor rock surfaces.

## Materials and methods

### Vouchers used for molecular analyses

For molecular genetic analyses, we used specimens from either archived herbarium vouchers or recently collected, uncatalogued vouchers. The age of the selected vouchers ranged from 20 years (in rare cases) to between a few months and <5 years (as in most cases). Newly acquired vouchers of the genus *Trapelia* were from North America and central and northern Europe, while new specimens of *Placopsis* came from New Zealand, Alaska, northern Europe, Asia (western China, Russian Far East) and South America (Chile; Table S1, Supporting information). As in Resl *et al*. (2015), *Placopsis* is circumscribed here to encompass *Aspiciliopsis* and *Orceolina*. We adopt provisional naming schemes for *Placopsis gelida* and *P. lambii* because beyond narrow geographic regions, neither name is currently being used in the sense of the type specimens (T. Spribille *et al*., in prep.). We also use several DNA samples from several new species to science, given provisional names in quotation marks, that shall be formally described elsewhere. In all, taxon sampling covered about half of the known species of both genera (Table S2, Supporting information). All voucher specimens were stored in a dry state at room temperature prior to the preparation for DNA extraction and morphometric measurements.

### DNA extraction, PCR and sequencing

Samples for DNA extraction were prepared by cutting off apothecia or excising soredia with a razor blade from the voucher specimens. In cases when only insufficient amounts of apothecia or soredia were available, we excised nonsorediate thallus material, avoiding substrate contamination. We excluded lichen material that showed signs of contamination with lichenicolous fungi or fungal parasites. All preparations were performed using a dissecting scope. We extracted total DNA using the QIAGEN DNeasy Plant Mini Kit Quick Start Protocol (Centrifugation Protocol), the E.Z.N.A. HP Plant DNA Mini Kit (Centrifugation Protocol for Fresh or Frozen Specimens) or the QIAGEN QIAamp DNA Investigator Kit (Protocol: Isolation of Total DNA from Tissues) following manufacturers’ instructions. Protocol modifications include the removal of the RNase step and increased (to 5 min) column ethanol evaporation times.

We used up to eight fungal loci, namely the internal transcribed spacer region (ITS), which consists of two parts and includes the strongly conserved 5.8S rRNA region, the nuclear small ribosomal subunit gene (SSU), the nuclear large ribosomal subunit gene (LSU) and the mitochondrial small ribosomal subunit gene (mtSSU), and four protein‐coding genes, namely DNA‐directed RNA polymerase II subunit RPB1 (RPB1), DNA‐directed RNA polymerase II subunit RPB2 (RPB2), DNA replication licensing factor MCM7 (MCM7) and translation elongation factor 1 alpha (EF1a). Primer pairs and annealing temperatures are shown in Table S3 (Supporting information). Illustra PuReTaq Ready‐To‐Go PCR Beads were used for PCR amplification. The PCR programs followed the general structure: initial denaturation at 95 °C for 5 min; 35 cycles—denaturation at 95 °C for 1 min, annealing for 1 min (temperature primer pair‐specific; see Table S3, Supporting information), extension at 72 °C for 1 min; final extension at 72 °C for 7 min; storage at 4 °C until further use. We conducted a higher number of PCR cycles and included touchdown PCR cycles when primer pair specificity was expected to be low, especially in the case of EF1a, RPB1 and RPB2 primer pairs. We visualized amplified DNA fragments using ethidium bromide or Midori Green (NIPPON Genetics EUROPE) as fluorescent dyes under UV light.

We purified PCR products using the Omega E.Z.N.A. Cycle Pure Kit Centrifugation Protocol, Agencourt AMPure XP Bead Cleanup or the QIAGEN QIAquick PCR Purification Kit following manufacturers’ protocols. In the case of double bands after PCR, bands were excised and purified using the Omega E.Z.N.A. Gel Extraction Kit (Spin Protocol) and sequenced separately. Automated Sanger sequencing was conducted on an ABI 3730xl by Microsynth (Switzerland). In the majority of cases, sequencing was performed only in one direction using either the forward or reverse PCR primer as sequencing primer (see Table S3, Supporting information) although in some cases, sequencing was performed in both directions. Base calling was performed using Chromas Lite 2.1.1 (Technelysium).

### Phylogenetic analyses

For analyses not requiring access to the original specimens, we added additional *Placopsis* sequences from GenBank to our data set (see Table S1, Supporting information). We performed multiple sequence alignment using mafft 7 (Katoh & Standley 2013). For non‐protein‐coding genes, we used the option *–genafpair*, which is particularly suitable when a large number of introns are present, as in our case. For the protein‐coding genes, we chose the option *–globalpair* to ensure that insertions or deletions and thus frameshifts are avoided. We performed 10 000 iterations for each gene. Aligned sequences were inspected and adjusted manually to ensure the quality of the alignment. We concatenated all eight loci using in‐house Python and UNIX shell scripts (Resl 2015; Resl *et al*. 2015). For the construction of molecular phylogenies, we used a total set of 126 vouchers and 7983 nucleotide positions and a combination of maximum‐likelihood (ML), Bayesian and Bayesian multispecies coalescent methods. Maximum‐likelihood analyses were performed using raxml 7.4.2 (Stamatakis 2006) as implemented in raxmlgui 1.3 (Silvestro & Michalak 2012) with GTR + Γ + I as substitution model. We enabled different substitution rates for each locus by setting one partition for each of the eight genes. Statistical node support was evaluated by calculating 1000 bootstrap replicates using the ML + rapid bootstrap algorithm. The tree was rooted with *Ainoa*,* Parainoa* and *Baeomyces* as outgroup taxa following a previous study (Resl *et al*. 2015).

We calculated Bayesian molecular phylogenies on the basis of the same data set as in the ML analysis, using three different approaches, namely mrbayes, beast and * beast. In mrbayes 3.2.1 (Ronquist *et al*. 2012), we chose the options lset nst = 6 and rates = invgamma, corresponding to a GTR + Γ + I substitution model. The choice of this substitution model does not reflect a subjective decision in mrbayes and also needs no validation using model testing software, as substitution model parameters and the number of parameters are sampled in the course of the MC^3^ run. We used uninformative priors at the default settings. We ran four parallel chains for 5 million generations, sampling every 500th generation. The chain mixing parameter was kept at the default value. We checked for convergence of the MC^3^ algorithm by examining stationarity of the posterior probability and likelihood using tracer 1.6 (Rambaut *et al*. 2014). We removed the first 50% of generations, corresponding to 5000 trees, as burn‐in. The posterior tree sample containing 5000 trees, after removal of burn‐in, was used to construct a consensus tree based on all compatible groups (contype = allcompat).

Next, we used beast 1.8.1 (Drummond *et al*. 2012) to construct a time‐calibrated, ultrametric tree in a Bayesian framework. To set up the xml file on which a beast run is based, we used the GUI beauti 1.8.1 (Drummond *et al*. 2012). We partitioned the data set into four protein‐coding and four noncoding genes, for which we allowed substitution rates to vary freely. The GTR + Γ + I model with four Γ categories was chosen as substitution model. We used an uncorrelated relaxed clock model with the default parameter values for ucld.mean and ucld.stdev. Because defining a root age is required in beauti 1.8.1, the age of the most recent common ancestor (MRCA) of all included samples was set to an approximate estimate of 200 Ma (Prieto & Wedin 2013; Beimforde *et al*. 2014) with a standard deviation of 20 Ma. However, age estimates were not incorporated in phylogenetic comparative and other analyses. The tree prior followed a Yule pure‐birth process (Yule 1924) using a random starting tree. We kept all other parameter values at default settings. We performed two separate runs and checked for potential topological conflict. Each was allowed to run for 10 million generations. Trees were sampled every 5000th generation. tracer 1.6 (Rambaut *et al*. 2014) was used to check for stationarity of the likelihood and posterior probability as well as to inspect effective sample size (ESS) values of parameters. A maximum clade credibility (MCC) tree was calculated from the last 5 million generations of the posterior sample.

Finally, we used * beast 1.8.1 (Drummond *et al*. 2012) to construct a time‐calibrated, ultrametric species tree from the same data set as before. * beast calculates a species tree using a multispecies coalescent process (Rannala & Yang 2003), which accounts for incomplete lineage sorting effects and can also better cope with topological conflict among loci. Again, we used beauti 1.8.1 (Drummond *et al*. 2012) to set up the xml file. Species were defined based on bGMYC clusters. We set the age of the MRCA to be the same as in the first analysis. Population size was kept constant. We divided the data set into eight partitions, four for the protein‐coding and four for the noncoding genes, and allowed substitution rates to vary freely among loci. However, gene trees were linked to improve convergence of the MCMC runs. GTR + Γ + I with four Γ categories was chosen as the substitution model for all genes. We used an uncorrelated relaxed clock model for the clock/branch rate, keeping the ucld.mean and ucld.stdev parameters at their default values. For the species tree prior, we selected a Yule pure‐birth process speciation model. All other parameters were kept at default values. The MCMC chain was set to 250 million generations. Trees were sampled every 2500th generation. We used tracer 1.6 (Rambaut *et al*. 2014) to ensure convergence and stationarity of the likelihood and posterior probability and to check ESS values of parameters. We calculated an MCC tree based on the posterior sample of trees from the last 50 million generations. Two separate runs were conducted and checked for topological conflict. Finally, we compared the phylogenetic trees constructed by all four methods for topological conflict.

### Species delimitation

To provide unbiased *Placopsis* species definitions for pGLMM analyses (see below), we employed the Bayesian mixed Yule‐coalescent method implemented in the bGMYC r package (Reid & Carstens 2012). bGMYC estimates species boundaries by integrating over phylogenetic uncertainty and model parameters of the Yule and coalescent model components in an MCMC approach. We ran the analysis on 100 random beast trees for 50 000 generations, a burn‐in of 10 000 and thinning parameter of 100. Results were summarized as heatmap of pairwise co‐assignment probabilities on the MCC tree. Additionally, we performed k‐medioid clustering implemented in the function pamk of the r package fpc. In pamk, we used average silhouette width as cluster criterion on the probability matrix obtained from bGMYC.

### Morphometric analyses

We measured thalline, cephalodial and hymenial volumes (proxies for mass) in all specimens used in the phylogenetic analyses (hymenium = apothecial core containing sexual spores; for morphological structures, see Fig. 1), apart from GenBank samples. The measurement of mean thallus surface area, while a logical and very visible measure of thallus size increase across evolution, was not undertaken because herbarium specimens usually include only few thalli, and these are often fragmented due to the need to chisel samples out of rock. Thallus thickness may also be a better proxy for lichen productivity as it is a measure independent of the examined surface area, analogous to biomass per area in vegetation science. All vouchers were measured in a dry state. First, we defined areas on each thallus using cardboard rings (radius = 0.5, 0.75, or 1.25 cm). In some cases, half or a quarter of the area defined by the ring was taken when the thallus was too small to fill the area. Then, we determined the number of cephalodia and apothecia (if present). Depending on the number of present cephalodia, we randomly selected up to seven (but, if present, at least two) for volume measurements, assuring randomness by choosing the closest cephalodium to the crosshairs of the reticule prior to adjustment of a specimen's position (i.e. without first looking into the objective). We repeated the latter procedure for every measurement after randomly moving the specimen on the dissecting scope stage without looking into the objectives. The higher the perceived variation in cephalodial volume was, the more measurements were taken. We performed measurements by bisecting the selected cephalodium and then determining its diameter and thickness using the calibrated micrometre disc in one of the objectives. We then applied either the volume formula for a cylinder (*V* = (diameter/2)^2^ × π × thickness) or, in rare cases, a hemisphere (*V* = 2/3 × (diameter/2)^3^ × π) (depending on cephalodial form). We performed the same procedure in the case of apothecia/hymenia. However, we measured the hymenia of at least five and, if present, up to 10 apothecia per specimen. For the hymenia, the volume formulas were either for a cylinder or, in rare cases, for a cone (one‐third the volume of a cylinder). Juvenile apothecia were excluded. To obtain the volumes per area, we calculated the arithmetic means and medians of cephalodial and hymenial volumes, multiplied by the number of cephalodia or apothecia, respectively, on the defined area of the thallus, and divided by the area as calculated using the formula (radius)^2^ × π.

As proxy for thallus volume per area, we determined thallus thickness at five random positions in the area defined by the cardboard rings. Again, we assured randomness using the above‐mentioned procedure. In cases when there was high perceived variation in thallus thickness, up to ten measurements were taken. Measurements were taken by excising small pieces of thallus using a razor blade. We calculated arithmetic means and medians of the thallus measurements for each specimen. In a predefined, fixed area of the thallus, thallus thickness constitutes a measure directly proportional to thallus volume in that area.

Substrates such as bryophytes, other lichens, fungi, conspicuous biofilms or decayed organic material are potentially nutrient enriched (Arróniz‐Crespo *et al*. 2014). To the extent thalli grow up and onto such substrates, this could positively affect the size of thalli, cephalodia and apothecia. We recorded the presence/absence of these substrates for each of the thallus measurements when measuring thallus thickness as described above, irrespective of the thickness of potentially nutrient‐rich substrates. Even when a potentially nutrient‐rich substrate was recorded in just one of the thallus measurements of a specimen, we counted the overall value of this variable as presence for the whole specimen (rare cases).

We calculated arithmetic means and medians of mean thallus thickness, mean hymenial volume and hymenial volume per area separately for *Placopsis* and *Trapelia*. Morphometric characters were log‐transformed (using the natural logarithm) to account for non‐normality. We then performed Welch's two‐sample *t*‐tests (Welch 1947) to compare morphometric character distributions between *Placopsis* and *Trapelia*. For mean thallus thickness and mean hymenial volume comparisons between *Placopsis* and *Trapelia*, we used one‐sided *t*‐tests (null hypothesis: *Placopsis* = *Trapelia*; alternative hypothesis: *Placopsis* > *Trapelia*). For hymenial volume per area, we used a two‐sided *t*‐test. All statistical analyses were performed in r (R Core Team 2015).

### Character mapping

We applied a ML approach to trace morphometric characters across the *Placopsis* and *Trapelia* phylogeny. For this purpose, we used a custom r script employing the packages phytools (e.g. function contMap; Revell 2012) and ape (Paradis *et al*. 2004; Popescu *et al*. 2012) to construct a continuous character map, which we then plotted onto the phylogeny of one of the two BEAST MCC trees. The plotted characters include the relative natural logarithms (between 0 and 1) of the mean thallus thickness along with mean volumes of cephalodia or volumes of cephalodia per area. We created phylogenetic and morphometric character coplots (i.e. combinations of trees and bar plots) using custom r scripts using the packages ape, adephylo (Jombart *et al*. 2010) and phylobase (Bolker *et al*. 2010; Hackathon *et al*. 2011). Plotted characters include the relative mean thallus thickness, mean hymenial volume and hymenial volume per area.

### Phylogenetic comparative analyses

We employed various statistical frameworks to explore the relationships among the four studied variables thallus volume, cephalodial volume, hymenial volume and the presence/absence of potentially nutrient‐rich substrate. The core application of these methods here was phylogenetic generalized least squares (PGLS; Grafen 1989), implemented using custom r scripts (R Core Team 2015) and the r packages ape (Paradis *et al*. 2004; Popescu *et al*. 2012) and nlme (Pinheiro *et al*. 2014). As all subsequent PGLS and pGLMM analyses included cephalodial volume as explanatory variable, we included only cephalodia‐bearing samples of *Placopsis* sensu stricto (i.e. excluding *P. roseonigra*;* n *=* *72). PGLS analyses were based on the MCC tree of one of the two *BEAST* runs, which did not differ with respect to topology. Samples other than from *Placopsis* sensu stricto were pruned from the tree. We modelled the phylogenetically dependent correlation using the four evolutionary correlation structures Brownian motion (Felsenstein 1985; Martins & Hansen 1997), Pagel's λ (Pagel 1999; Freckleton *et al*. 2002), Ornstein‐Uhlenbeck (OU; Uhlenbeck & Ornstein 1930; Martins & Hansen 1997) and Blomberg's *ACDC* (Blomberg *et al*. 2003), which were compared by Akaike's information criterion (AIC; Akaike 1974; Sakamoto *et al*. 1986). Model parameters were estimated using maximum likelihood, except for the γ parameter in Blomberg's ACDC model, which was fixed at 0.2, 0.6, 1.4, 1.8 or 2.2 (1.0 was omitted as this would render the model equivalent to a Brownian motion model). Because of the different numbers of specimens per species cluster in the phylogenetic data set, we weighted the effects of each species on regression by the inverse of the number of specimens of the bGMYC cluster in question using a weighting matrix. In total, we compared four unweighted and four weighted models using AIC. For each of these models, we determined the significance of the intercept and slope of the regression using adjusted *t*‐tests included in the nlme package. We performed PGLS regression with mean thallus thickness as dependent and mean cephalodial volume as explanatory variable (model 1). Additionally, we split the data according to the binary variable presence/absence of a potentially nutrient‐rich substrate, and again the relationship between mean thallus thickness and mean cephalodial volume was examined, thus obtaining models 2 and 3. We repeated PGLS analyses using the cephalodial volume per area as explanatory variable (models 4–6, respectively). We also conducted PGLS analyses using mean hymenial volume as dependent variable and mean cephalodial volume or cephalodial volume per area as explanatory variables (models 7 and 8) as well as using hymenial volume per area as dependent variable and the same explanatory variables as before (models 9 and 10). Due to moderate deviations from the normality assumption, we log‐transformed all morphometric variables using the natural logarithm.

In the second part of the phylogenetic comparative analyses, we used Bayesian phylogenetic generalized linear mixed‐effects models (pGLMM; Hadfield & Nakagawa 2010) to divide variance into a within‐ and a between‐species component, which is not possible using PGLS. pGLMM analyses were implemented using custom r scripts and by making use of the r packages ape and mcmcglmm (Hadfield 2010). We employed the MCC species tree of one of the two * beast runs, which did not differ with respect to topology. Again, we used only cephalodia‐bearing *Placopsis* samples in these analyses. mcmcglmm uses a Bayesian Markov chain Monte Carlo algorithm to obtain posterior means, credibility intervals and *P*‐values (pMCMC‐values) for the effect of the explanatory variable on the dependent variable, divided into a between‐species and within‐species as well as a residual component. We conducted pGLMM analyses using models 1, 4, as well as models 7–10, that is all of the above models that did not discriminate between the presence/absence of nutrient‐rich substrate. Because of deviations from normality, we log‐transformed the morphometric variables using the natural logarithm prior to MCMC runs. The Markov chain ran for 5 million generations and was sampled every 500th generation. We discarded the first 100 000 generations based on the posterior probability and likelihood trace plots of the mcmcglmm output. PGLS and pGLMM figures were produced using the r package ggplot2 (Wickham & Chang 2009).

## Results

### *Trapelia*–*Placopsis* phylogeny and intergeneric relationships

**Figure 2:**
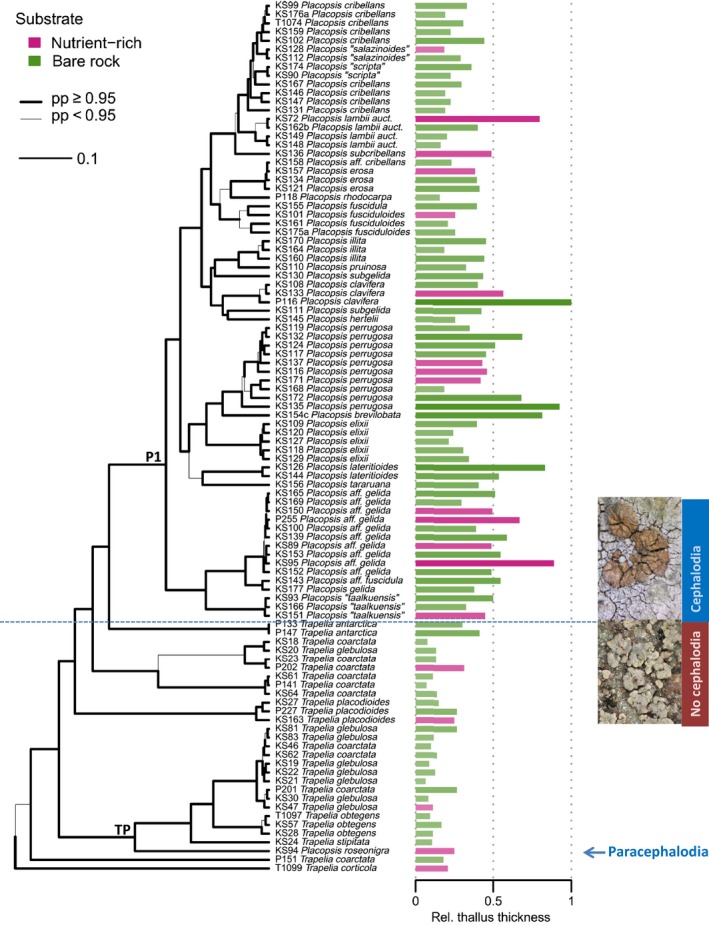
Distribution of mean thallus thickness over beast MCC phylogeny. The presence (purple) or absence (green) of potentially nutrient‐rich substrate is shown using colour‐coding in the bar plot. The transition from *Trapelia* to *Placopsis* sensu stricto is indicated by a dashed blue line. Branch thickness corresponds to posterior probability (pp). The scale bar indicates the number of substitutions per nucleotide site. lower picture: *T. glebulosa* (KS47); upper picture: *P. gelida* (KS177); TP:* Trapelia* and *P. roseonigra* clade; P1: *Placopsis* s.str. clade.

In the mrbayes analysis, the average standard deviation of split frequencies stabilized at 0.0066 after 5 million generations. In the beast and * beast analyses, the ESS of the likelihood converged at 191 and 1063, respectively, at the end of the runs that were used for further analyses (‘run1’ in Dryad). Species delimitation results are reported in Fig. S1 and Table S1 (Supporting information). In all performed phylogenetic analyses, the intergeneric relationships between *Placopsis* and *Trapelia* remained constant with high statistical node support (pp = 1.00; see Fig. 2 for pruned beast tree, Fig. S2, Supporting information, for species tree and Fig. S3, Supporting information, for complete beast tree). The main clade of *Placopsis* (node P1; Fig. 2) was nested within *Trapelia*, as in the study by Resl *et al*. (2015). The second evolutionary joint between *Trapelia* and *Placopsis* is a product of the placement of the enigmatic *P. roseonigra* at the base of one clade of *Trapelia* (thus forming node TP in Fig. 2), strictly rendering the genus *Placopsis* polyphyletic. *Trapelia*, however, has to be considered paraphyletic even in the absence of *P. roseonigra* because the *Placopsis* main clade (node P1) is nested within *Trapelia*.

### Morphometric comparisons between *Trapelia* and *Placopsis*

The arithmetic mean of mean thallus thickness was 152% higher (median: 196% higher) and the mean of mean hymenial volume 847% higher (median: 719% higher) in *Placopsis* than in *Trapelia* (see Table 1). The difference in arithmetic means was highly significant (i.e. *P* ≪ 0.001). While the median of the hymenial volume per area was slightly higher in *Trapelia* than in *Placopsis*, the arithmetic mean of the same character was slightly lower in *Trapelia*. However, hymenial volume per area was not significantly different between the two genera (Table 1).

**Table 1:** Basic descriptive statistics and *t*‐test results for the *Placopsis*‐*Trapelia* comparison

Statistical measure	Mean thallus thickness (mm)	Mean hymenial vol. (mm^3^)	Hymenial vol. per area (mm^3^/cm^2^)
Mean in *Placopsis*	0.361	0.675	0.717
Mean in *Trapelia*	0.143	0.071	0.695
Median in *Placopsis*	0.340	0.472	0.421
Median in *Trapelia*	0.115	0.058	0.480
Welch's *t*‐test results	*t* = 8.78; d.f. = 47.2; *P* = 8.76 × 10^−12^	*t* = 9.07; d.f. = 60.7; *P* = 3.37 × 10^−13^	*t* = 0.2079; d.f. = 47.2; *P* = 0.836

For Welch's *t*‐tests, the natural logarithm of each morphometric character was taken.

### Morphometric comparisons based on substrate

Arithmetic means and medians of mean thallus thickness were higher in specimens growing on potentially nutrient‐rich substrates such as bryophytes or fungi than in specimens growing on bare rock (see Table S4, Supporting information). This was true for both the pooled comparison (*Placopsis* and *Trapelia* combined) and the separate comparisons (within *Placopsis* and within *Trapelia*). Welch's *t*‐tests also revealed significantly higher mean thallus thicknesses in specimens growing on potentially nutrient‐rich substrates than in specimens growing on bare rock in all comparisons (*P *<* *0.05; Table S4, Supporting information).

### Distribution of thallus and hymenial volumes across phylogeny

The ML character maps showed a clear average increase in volumes at the transition from *Trapelia* to *Placopsis*. This became particularly clear in the mean thallus thickness character map (Fig. 2), where the mostly high values in *Placopsis* pronouncedly contrasted with the typically low values in *Trapelia*. In both *Trapelia* and *Placopsis*, specimens growing on potentially nutrient‐rich substrates clearly showed higher values of mean thallus thickness than specimens growing on bare rock (Fig. 2). High values of mean thallus thickness also coincided with high mean cephalodial volumes and cephalodial volumes per area (character maps: Figs S4 and S5, Supporting information). The strong contrast in volumes between *Placopsis* and *Trapelia* also became evident when looking at the mean hymenial volume coplot (Fig. S6, Supporting information), but this clear difference disappeared in the case of the hymenial volume per area coplot (Fig. S7, Supporting information).

### Correlation of thallus and cephalodial volume

**Figure 3:**
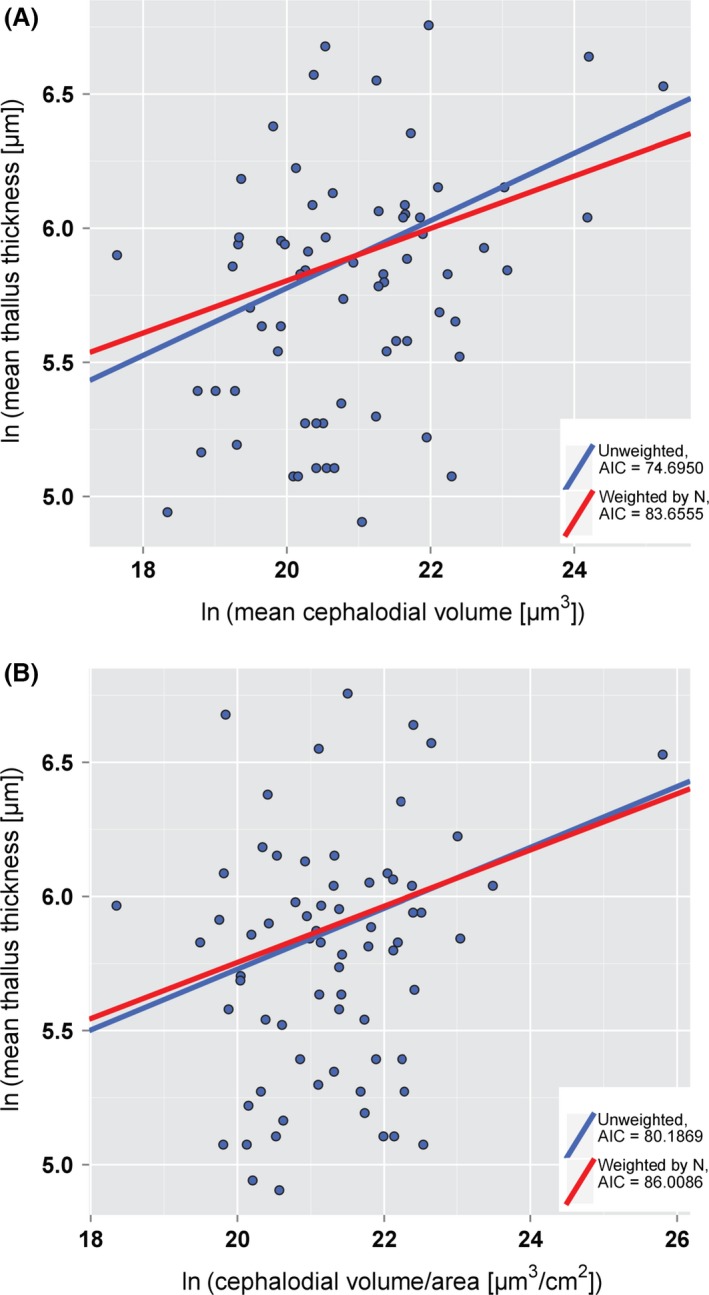
PGLS plot of mean thallus thickness against cephalodial volume. (A) The natural logarithm of mean thallus thickness is plotted against the natural logarithm of mean cephalodial volume. (B) The natural logarithm of mean thallus thickness is plotted against the natural logarithm of cephalodial volume per area. Blue line: PGLS regression line without weighting by sample size per bGMYC cluster; red line: PGLS regression line after weighting by sample size per bGMYC cluster.

In all PGLS analyses, the Pagel's λ model ranked highest according to the AIC. In all of the following, we will report relationships in the order‐dependent variable: explanatory variable. Within the genus *Placopsis* sensu stricto (i.e. excluding *P. roseonigra*), a highly significant (*P *<* *0.001) positive relationship between mean thallus thickness and mean cephalodial volume was detected (unweighted model ranked highest; slope *b *=* *0.13 ± 0.03 SE; see Fig. 3 and Table 2). The phylogenetic signal in this relationship was high (Pagel's λ = 0.63 ± 0.33 C.I.). The positive relationship between mean thallus thickness and cephalodial volume per area was also significant (*P *<* *0.01), albeit with a slightly lower slope (unweighted model ranked highest; *b *=* *0.11 ± 0.04 SE; Fig. 3 and Table 2). Phylogenetic signal for the latter relationship remained in the same order of magnitude (Pagel's λ = 0.56 ± 0.39 C.I.).

**Table 2:** Results of PGLS analyses with mean thallus thickness as dependent variable

Explanatory variable	Mean cephalodial volume		Cephalodial volume per area	
Parameter	Intercept *a*	Slope *b*	Intercept *a*	Slope *b*
Value	3.26	0.13	3.46	0.11
Std. error	0.71	0.03	0.92	0.04
*t*‐value	4.57	3.77	3.76	2.66
*P*‐value	0.0000	0.0003	0.0004	0.0097

Results are shown for unweighted regressions. All morphometric variables were transformed using the natural logarithm prior to analyses. Total d.f. = 70; residual d.f. = 68.

In the separate analysis where we only used specimens growing on nutrient‐rich substrates, the relationship between mean thallus thickness and mean cephalodial volume was similar to the relationship when using all specimens bearing cephalodia (model weighted by *N* ranked highest; slope *b *=* *0.11 ± 0.05 SE; see Fig. S8 and Table S5, Supporting information). Phylogenetic signal was much lower than in the above PGLS analyses (Pagel's λ = 0.04 ± 0.60 C.I.). PGLS results for the relationship between thallus thickness and cephalodial volume per area in specimens growing on nutrient‐rich substrates were similar (model weighted by *N* ranked highest; slope *b *=* *0.12 ± 0.09 SE; Fig. S8 and Table S5, Supporting information). However, phylogenetic signal was higher in the latter relationship (Pagel's λ = 0.21 ± 0.68 C.I.).

When using only specimens growing on potentially nutrient‐poor substrate (i.e. growing on bare rock surface) in PGLS analyses, the relationship between mean thallus thickness and mean cephalodial volume was similar to the results obtained using all cephalodia‐bearing specimens (unweighted model ranked highest; slope *b *=* *0.11 ± 0.05 SE; see Fig. S9 and Table S6, Supporting information). The same is true for the obtained ML estimate of the phylogenetic signal (Pagel's λ = 0.58 ± 0.38 C.I.). However, the relationship between mean thallus thickness and cephalodial volume per area was slightly weaker when using this subset of the data than when using all cephalodia‐bearing specimens (unweighted model ranked highest; slope *b *=* *0.08 ± 0.06 SE; Fig. S9 and Table S6, Supporting information). This was also the case with the phylogenetic signal (Pagel's λ = 0.50 ± 0.41 C.I.).

For the relationship between mean thallus thickness and mean cephalodial volume, pGLMM results revealed similar positive effect sizes and significances of the within‐species component (slope *b *=* *0.12; pMCMC = 0.0126; see Table 3) and the between‐species component (slope *b *=* *0.12; pMCMC = 0.0267; Table 3). In the case of the relationship between thallus thickness and cephalodial volume per area, only the within‐species component showed a significant positive relationship (slope *b *=* *0.11; pMCMC = 0.0347; Table 3), but the posterior mean of the slope was similar in the between‐species component (slope *b *=* *0.10; pMCMC = 0.2312; Table 3).

**Table 3:** Results of pGLMM analyses with mean thallus thickness as dependent variable

Explanatory variable	Mean cephalodial volume		Cephalodial volume per area	
Component	Between species	Within species	Between species	Within species
Posterior mean (slope *b*)	0.12	0.12	0.10	0.11
Lower 95% credibility interval	0.02	0.03	−0.07	0.01
Upper 95% credibility interval	0.23	0.22	0.27	0.21
*P*‐value (pMCMC)	0.0267	0.0126	0.2312	0.0347

All morphometric variables were transformed using the natural logarithm prior to analyses. *n *=* *72.

### Correlation of hymenial and cephalodial volume

PGLS did not reveal a significant relationship between the morphometric characters mean hymenial volume and mean cephalodial volume, despite a positive regression slope (see Fig. S10 and Table S7, Supporting information). Phylogenetic signal of this relationship was high (Pagel's λ = 0.72 ± 0.41 C.I.). In strong contrast to the above regressions, the relationship between mean hymenial volume and cephalodial volume per area was even moderately negative and almost significant (Fig. S10 and Table S7, Supporting information). Phylogenetic signal was high but slightly lower than in the previous regression (Pagel's λ = 0.63 ± 0.45 C.I.).

Despite the observed moderately positive relationship between hymenial volume per area and mean cephalodial volume, the regression slope was clearly nonsignificant (see Fig. S11 and Table S8, Supporting information). The phylogenetic signal of this relationship was weak (Pagel's λ = 0.23 ± 0.39 C.I.). Hymenial volume per area and cephalodial volume per area showed a moderately negative but clearly nonsignificant relationship (Fig. S11 and Table S8, Supporting information). The phylogenetic signal remained in the same order of magnitude as in the previous regression (Pagel's λ = 0.18 ± 0.38 C.I.).

pGLMM analyses revealed a positive and negative relationship between mean hymenial volume and mean cephalodial volume in the within‐ and between‐species component, respectively (slope *b *=* *0.37 and −0.17, respectively; see Table S9, Supporting information), albeit without being significant (pMCMC = 0.0553 and 0.4273, respectively) and with high variances. In contrast, the relationship between mean hymenial volume and cephalodial volume per area of both the within‐ and between‐species component was negative (slope *b *=* *−0.18 and −0.52, respectively; Table S9, Supporting information). Again, the regression slopes of both components showed high variance and were clearly nonsignificant (pMCMC = 0.3137 and 0.1743, respectively).

The pGLMM analysis using hymenial volume per area and mean cephalodial volume showed a stronger relationship in the within‐ than in the between‐species component (slope *b *=* *0.33 and 0.12, respectively; see Table S10). Also in this case, the regression slopes of both components showed high variances and were far from being significant (pMCMC = 0.3794 and 0.6441, respectively). The results of pGLMM regression of hymenial volume per area and cephalodial volume per area also showed high variances of the within‐ and between‐species components (Table S10). While the posterior mean of the slope of the within‐species component was only slightly negative, the same parameter was highly negative when looking at the between‐species component (slope *b *=* *−0.03 and −0.49, respectively). However, the slopes of both components were clearly nonsignificant (pMCMC = 0.9320 and 0.2288, respectively).

## Discussion

The *Trapelia*–*Placopsis* clade encompasses a diverse but monophyletic group of lichen‐forming fungi including 18 described species in the precyanobacterial *Trapelia* clades distributed in temperate parts of both hemispheres, and an estimated 62 species in the cyanobacteria‐associated *Placopsis* clades mostly in cold, high‐latitude regions, many restricted to New Zealand and the Subantarctic (Lamb 1947). Increasing taxon sampling has provided continuously improving resolution of the nature of evolution in this group, from reciprocal monophyly with *Trapelia* in single‐ or three‐locus gene phylogenies (Poulsen *et al*. 2001; Schmitt *et al*. 2003) to a paraphyletic *Trapelia*–*Placopsis* phylogeny in an eight‐locus sample with more species (Resl *et al*. 2015). For our present study, the largest to date for this group, we included more than five times as many *Placopsis* vouchers as Resl *et al*. (2015) with the aim to provide a morphometric analysis of thallus and apothecial size metrics both within and among multiple species with every morphometric voucher anchored in an eight‐locus phylogeny. Several species are included here for the first time, and our study is the first to show that *Placopsis roseonigra*, a rare North Pacific mountain species (Brodo 1995) first sequenced here, acquired cyanobacteria independently and is paraphyletic to the rest of *Placopsis*. Together with previously sequenced species, we now have DNA of 31 named species of *Placopsis*, half the known species of the genus. Filling the sampling gaps in *Placopsis* and *Trapelia,* some of which have not been rediscovered since their original description, will require field work in remote regions, mainly in Australasia and Chile, which was beyond the scope of this study.

While we could not infer a direct causal relationship, the strong positive correlation with thallus size at the transition from *Trapelia* to *Placopsis*, as well as the sustained pronounced correlation we found in PGLS and pGLMM analyses between cephalodial volume and thallus thickness after cyanobacterial acquisition, is a strong indication for a role of cyanobacteria and atmospheric nitrogen fixation in thallus size increases. Ecophysiological studies indicate that a burst of additional N directly translates into measurable changes in thallus metabolism. Elevated rates of nitrogenase activity, measured as acetylene reduction activity, have been documented from *Placopsis gelida* from Iceland (Crittenden 1975) and from three species of *Placopsis* in southern Chile (Raggio *et al*. 2012), in the latter case strongly positively correlating with N content and maximum photosynthetic rate of the green alga. Cephalodia are, however, not the sole source of additional nutrients nor is cephalodial volume the sole predictor of thallus thickness. We also found that thalli that overgrow potentially nutrient‐rich substrates such as bryophytes or fungi were thicker, on average, than those that do not. This form of enrichment may, however, likewise trace back to nitrogen‐fixing cyanobacteria living in bryophyte mats (Arróniz‐Crespo *et al*. 2014). For lichens growing on potentially nutrient‐rich substrates, we could not observe a breakdown of correlation, suggesting that increased nutrient supply translates into even larger thalli or suggesting that other factors such as possible P limitations on nitrogen fixation may likewise be expected to correlate with thallus size (Crittenden *et al*. 1994; Reed *et al*. 2007). However, sample size for lichens growing on nutrient‐rich substrates is low, and thus, care should be taken when drawing conclusions from this subset of the data.

It could be argued that the sharp increase in the number of species after acquisition of cyanobacteria constitutes an adaptive radiation. Current estimates put the number of described species in the pre‐acquisition *Trapelia* clades at about 18 and postacquisition *Placopsis* clades at 62 (Table S2, Supporting information), a ratio mirrored in our bGMYC estimates (13/39). The number of described species is constantly being revised upwards (*Trapelia*: Kantvilas *et al*. 2015; *Placopsis*: Galloway 2013) and objective estimates based on a complete molecular sampling of all described taxa, if that were possible, would likely be even higher. In addition to the apparent speciation burst, *Placopsis* species almost invariably occur in cold, wet climates (maritime to hypermaritime, boreal to arctic; Lamb 1947), a narrower range than in *Trapelia*, which spans dry‐to‐wet and temperate‐to‐arctic climates (Hertel 1970). Within the regions with highest *Placopsis* diversity, such as New Zealand and Chile, there is clear evidence that species have adapted to niches such as bare rock surfaces (e.g. *P. hertelii*), gravel bars of rivers (*P. trachyderma*), silt in glacial forelands (*P. pycnotheca*) and stabilizers of dry, bare soil banks, as along road cuts (*P. clavifera*; Ullmann *et al*. 2007; Raggio *et al*. 2012; Galloway 2013). All of these characteristics (ecological release, rapid speciation, morphological diversification) are classic hallmarks of an adaptive radiation (Gavrilets & Vose 2005; Yoder *et al*. 2010).

### Small thalli are small canvasses for exhibition of phenotype

A second marked transition concomitant with size increase in *Placopsis* is the increase in distinct morphological features that can be used to distinguish species. This subjective but important factor is reflected in the fact that nearly every *Placopsis* bGMYC cluster was previously named independent of, and almost invariably only later validated by, molecular data (Galloway 2013). This is not the case with *Trapelia*, where we recovered no fewer than seven bGMYC clusters filed under identifications of two species, *T. coarctata* and *T. glebulosa*, long considered to contain an intractable knot of microvariation for classical systematists (Hertel 1973; Brodo & Lendemer 2015). The term cryptic species has been used for similar cases where systematists have overlooked or misjudged characters and species‐level clades were first identified by molecular methods (Crespo & Pérez‐Ortega 2009). Some authors have claimed that these species undergo ‘morphological stasis’ or even that there is ‘a selective advantage of maintaining a specific phenotype’ (Lumbsch & Leavitt 2011). For one thing, this is a contradiction in terms: invariability of a specific phenotype leaves nothing on which selection can act. Although never explicitly stated by lichenologists, evolution without phenotypic divergence would require an alternative to speciation through natural selection or imply neutral speciation (Baptestini *et al*. 2013). Neutral speciation in lichenized fungi is a conceivable possibility but is unlikely to be common due to widespread sympatry, and would be difficult to prove. A more parsimonious explanation is that classical lichen systematists are making use of only a small fraction of the available toolbox to characterize the fungal phenotype. In the case of lichenized fungi, as with all fungi, selective advantages may be achieved through subtle biochemical shifts in energy storage (e.g. constitution of polymer matrices, lipid chemistry). Yeast researchers working with simple, seemingly identical, ellipsoid cells were confronted with similar issues over 100 years ago and found ways to characterize species, for example by describing their ability to utilize exogenous compounds (Barnett 2004). The phenomenon of ‘cryptic speciation’ may be as much a reflection of how much energy a science invests in characterization of phenotype than of any biological process.

Notwithstanding these caveats, it stands that the larger a thallus can be, the larger a canvas exists on which even subtle changes in internal metabolism will be betrayed by telltale morphology. One could argue that a combination of resource limitation and obligate association with nutrient‐poor acidic rock surfaces leaves most *Trapelia* lineages with an extremely reduced canvas on which only two motifs are repeatedly manifested: a filmy thallus (all forms that have been called ‘*coarctata*’) and an areolate thallus (forms that have been called ‘*glebulosa*’). A similar phenomenon can be observed in other major groups of obligately rock‐dwelling crust lichens. Species of *Porpidia*, which obligately colonize similar acidic rock surface microsites to *Trapelia*, likewise oscillate within a narrow range of thallus and fruiting body motifs, leading to species that are first detected by DNA sequencing (Orange 2014). The same general pattern, with variations on the theme, repeats itself in rock‐dwelling species of *Protoparmelia* (Singh *et al*. 2015), within *Tephromela* (Muggia *et al*. 2014), and within the *Sarcogyne*–*Acarospora* complex (Westberg *et al*. 2015). For the smallest thalli and simplest body plans (e.g., round apothecia, ellipsoid ascospores) the ‘reduced canvas’ offers few handles to characterize morphology at the scale at which most lichen systematists work. Constraints on size and the associated possibility of displaying variation may be less pronounced in lineages not bound to a single substrate, possibly because they are trophically more flexible (P. Resl and T. Spribille, unpublished).

### Fruiting body investment shifts from ‘many small’ to ‘few large’

One unexpected and initially counterintuitive result was the discrepancy between increases in mean hymenial volume per apothecium on the one hand, and hymenial volume per surface area on the other. While the mean hymenial volume per apothecium showed a pronounced increase at the transition to *Placopsis*, the hymenial volume per area did not differ substantially between the two genera; in other words, the hymenial mass divided over many small apothecia in *Trapelia* became consolidated into few apothecia in *Placopsis*. Apothecial abundance can be related to environmental stress or parasites (Fahselt *et al*. 1989; Seymour *et al*. 2005), but our sampled thalli were healthy. Hymenial volume per area as a proxy for investment in sexual reproduction on the level of the lichen as a whole appears to scale allometrically with thallus size, where additional resources in the form of higher nitrogen input are allocated to asexual rather than sexual structures. The much higher values of mean hymenial volume per apothecium, by contrast, could either indicate higher investment in single spores, or portend a higher number of spores per apothecium. Alternatively, the number and size of spores may be ± constant in both genera, and large apothecia may merely be more efficient than small ones, or, alternatively, relocation of nutrients may be easier in large, contiguous thalli. In this case, the reason for *Trapelia* not producing large apothecia could be the low productivity of the thallus and its discontinuity. We are not aware of comparable observations of this phenomenon from lichens.

### The *Trapelia–Placopsis* system as a case study of size increase evolution

The evolution of *Placopsis* from within *Trapelia* is only one of several abrupt thallus size transitions, from small, biofilm‐like crusts to large, showy, radiating thalli, that happened in lichen evolution. Several of the most important origin events for large lichen thalli—those of the Parmeliaceae, of *Cladonia* and of *Stereocaulon*, and the radiation of Peltigerales—took place in the Cretaceous (~150–50 Ma BP; Beimforde *et al*. 2014) and their exact origins will be challenging to reconstruct. What we do know, however, based on living ancestors and the majority of diversity in Lecanoromycetes and their photobionts, is that thallus size and volume must have increased in some cases by orders of magnitude. Until now, no evolutionary hypotheses have been advanced on what innovations made this possible. The *Trapelia*–*Placopsis* transition is relatively more recent and provides a window into how some of these transitions may have unfolded. As our present study shows, the mere symbiosis with an algal symbiont is not enough for *Trapelia* to form a thallus any larger than a few millimetres in diametre, but incorporation of cyanobacteria in thalline cephalodia coincides with thicker thalli and over ninefold more voluminous hymenia per apothecium.

Thallus size increases occurred multiple times over lichen evolution, and we expect that each case involved unique factors. However, we consider it noteworthy that cyanobacteria were close to the points of origin in several ancient small‐to‐large transitions not studied in detail here. The architecturally diverse macrolichens of the genus *Cladonia* share a common ancestor with the cephalodial, partially crustose members of *Pilophorus* (Stenroos *et al*. 2002; Miadlikowska *et al*. 2014); the large fruticose species of *Stereocaulon,* which possess cephalodia, arose from crustose ancestors that lacked cephalodia, including *Hertelidea* and *Lepraria* (Högnabba *et al*. 2014; Miadlikowska *et al*. 2014); and species of the order Peltigerales, which include some of the largest lichenized thalli known to science, such as *Lobaria pulmonaria*, arose through acquisition of cyanobacteria from a common ancestor with crustose Lecideaceae, although the exact relationships are still poorly resolved (Miadlikowska *et al*. 2014). It is true that not all cyanobacterial lichens are large, and not all lichens that lack cyanobacteria are small. It is, however, inescapable that a symbiotic or genetic innovation is required for a lichen thallus to undergo a manifold size increase in evolution, and in the case of *Placopsis*, evidence points strongly to a correlation with cyanobacteria. Resolving the evolutionary origin of macrolichens will require higher resolution of microbial players in the symbiosis, fully resolved phylogenies and improved sampling of basal symbionts, many of which are rare.

K.S. and T.S. designed the study. K.S. and T.S. wrote the manuscript with contributions from P.R. K.S. performed laboratory work with contributions from P.R. and T.S. K.S. performed morphometric measurements, statistical, phylogenetic and phylogenetic comparative analyses. P.R. performed bGMYC analyses. All authors contributed to figures and tables.

## Data accessibility

DNA sequences are available in GenBank under Accession nos KU844340–KU844777. Phylogenetic trees and the DNA alignment are available from the Dryad Digital Repository: http://dx.doi.org/10.5061/dryad.03692. See Table S11 for the morphometric character matrix.

## Supporting information


**Fig. S1** bGMYC probability map of species assignments.

**Fig. S2** * beast MCC species tree based on bGMYC species clusters.

**Fig. S3** Complete beast MCC tree.

**Fig. S4** Continuous character map—mean thallus thickness (left) and mean cephalodial volume (right).

**Fig. S5** Continuous character map—mean thallus thickness (left) and cephalodial volume per area (right).

**Fig. S6** Distribution of relative mean hymenial volume over beast MCC phylogeny.

**Fig. S7** Distribution of relative hymenial volume per area over beast MCC phylogeny.

**Fig. S8** PGLS plot of mean thallus thickness against cephalodial volume for specimens growing on potentially nutrient‐rich substrate.

**Fig. S9** PGLS plot of mean thallus thickness against cephalodial volume for specimens not growing on potentially nutrient‐rich substrate.

**Fig. S10** PGLS plot of mean hymenial volume against cephalodial volume.

**Fig. S11** PGLS plot of hymenial volume per area against cephalodial volume.

**Table S1** Information on voucher specimens and GenBank accessions.

**Table S2** *Placopsis* and *Trapelia* species synonym list.

**Table S3** PCR and sequencing primers used in this work.
**Table S4** Basic descriptive statistics and *t*‐test results for the *Placopsis*–*Trapelia* comparison based on substrate type.
**Table S5** Results of PGLS analyses with mean thallus thickness as dependent variable. Only specimens growing on potentially nutrient‐rich substrate were included.
**Table S6** Results of PGLS analyses with mean thallus thickness as dependent variable. Only specimens growing on potentially nutrient‐poor substrate (bare rock surface) were included.
**Table S7** Results of PGLS analyses with mean hymenial volume as dependent variable.
**Table S8** Results of PGLS analyses with hymenial volume per area as dependent variable.
**Table S9** Results of pGLMM analyses with mean hymenial volume as dependent variable.
**Table S10** Results of pGLMM analyses with hymenial volume per area as dependent variable.

**Table S11** Morphometric character matrix.
