# Supplementary material for: Escape from the cryptic species trap: lichen evolution on both sides of a cyanobacterial acquisition event
Source: Mol Ecol. 2016 May 11;25(14):3453–68. doi: 10.1111/mec.13636 (PMC5324663; doi:10.1111/mec.13636)
Supplement: Supplementary file 1 — Fig. S1 bGMYC probability map of species assignments. [file MEC-25-3453-s001.pdf]

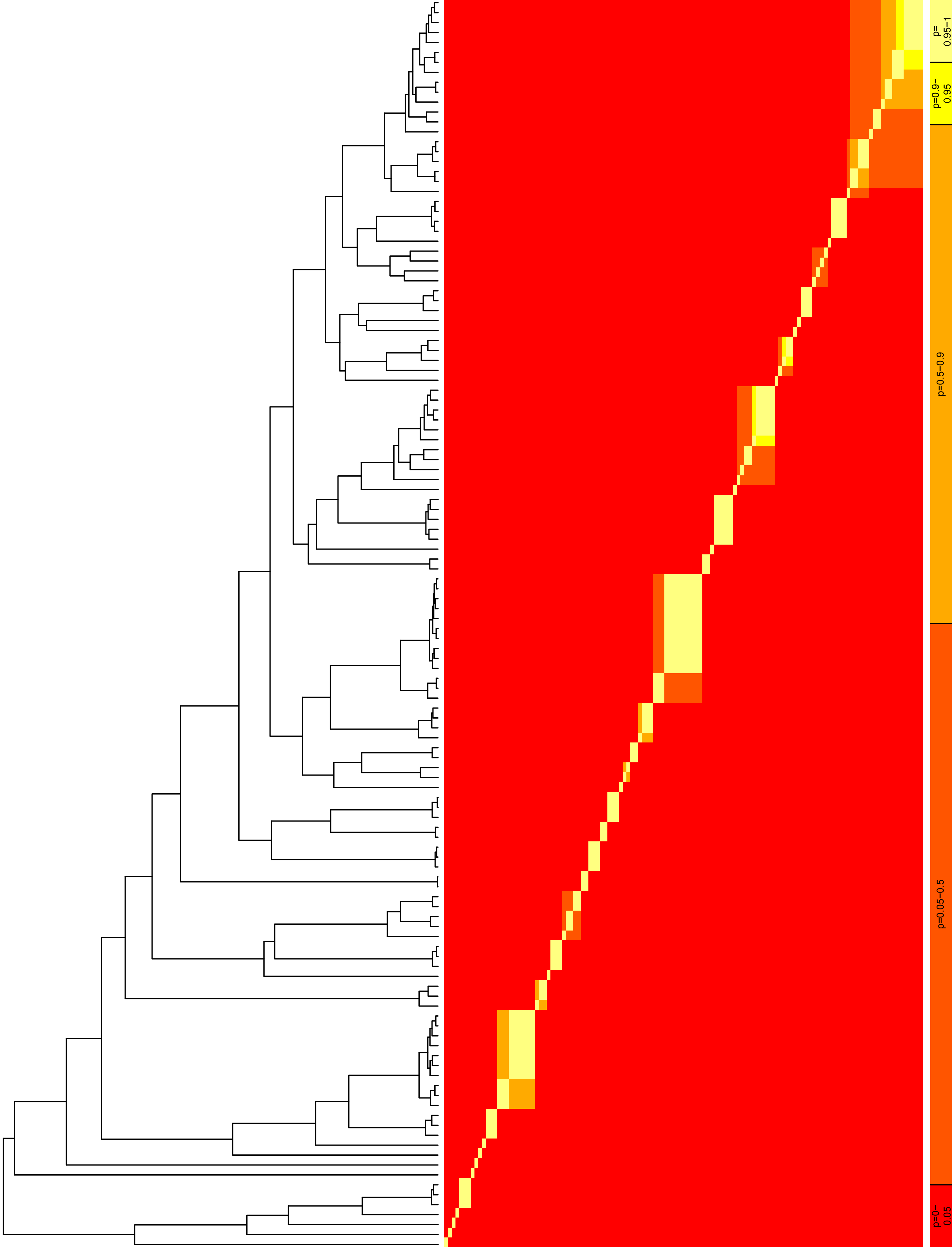

Fig. S1 – *bGMYC* probability map of species assignments.
The heatmap on the right showing posterior probabilities of species assignments is projected onto the MCC tree based on the *BEAST* posterior tree sample used for *bGMYC* analyses.
