# Supplementary material for: Escape from the cryptic species trap: lichen evolution on both sides of a cyanobacterial acquisition event
Source: Mol Ecol. 2016 May 11;25(14):3453–68. doi: 10.1111/mec.13636 (PMC5324663; doi:10.1111/mec.13636)
Supplement: Supplementary file 2 — Fig. S2 * beast MCC species tree based on bGMYC species clusters. [file MEC-25-3453-s002.pdf]

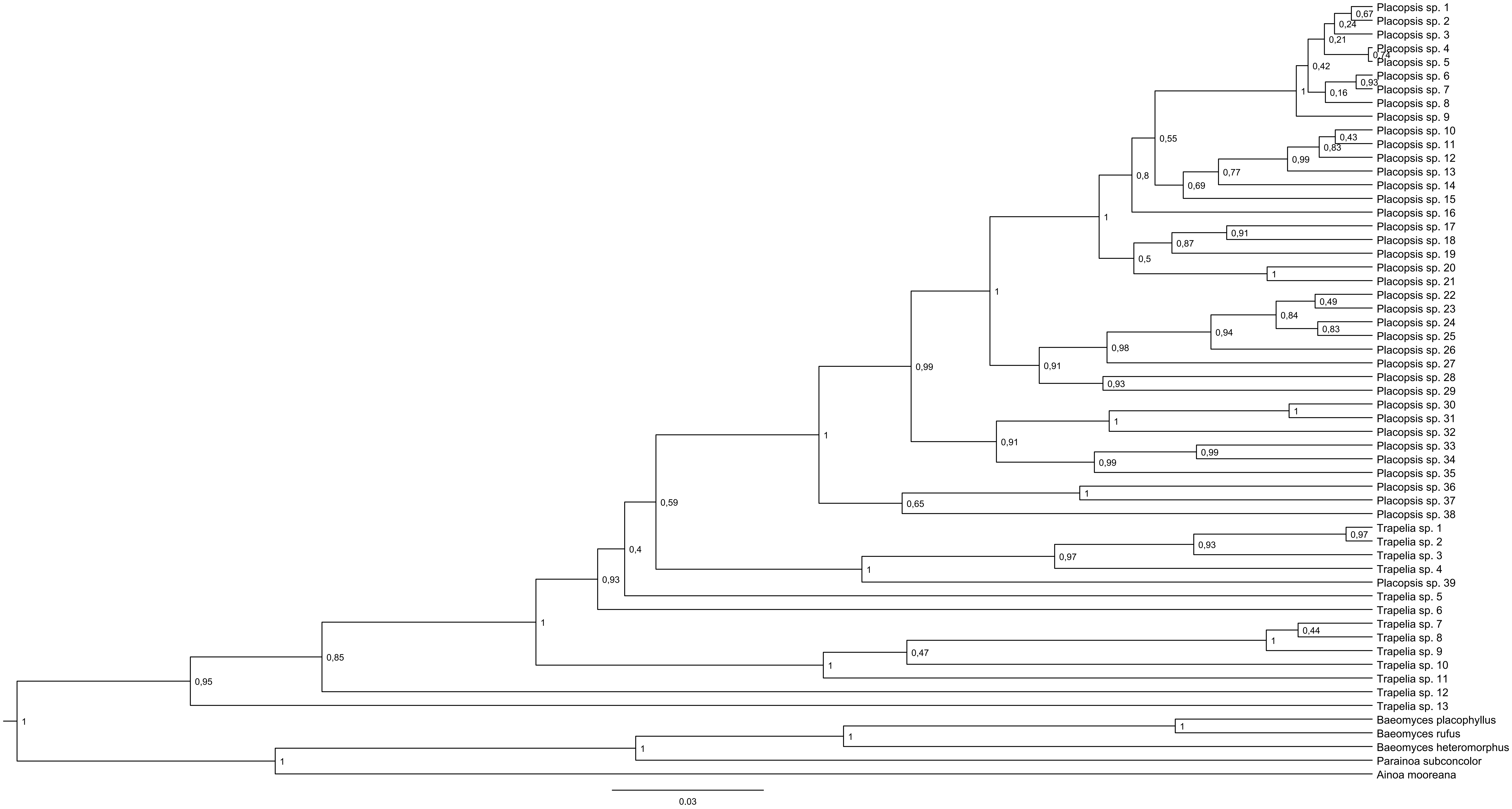

Fig. S2 – **BEAST* MCC species tree based on *bGMYC* species clusters.
Node support is indicated by posterior probabilities (*pp*). The scale bar indicates the number of substitutions per nucleotide site.
