# Supplementary material for: Escape from the cryptic species trap: lichen evolution on both sides of a cyanobacterial acquisition event
Source: Mol Ecol. 2016 May 11;25(14):3453–68. doi: 10.1111/mec.13636 (PMC5324663; doi:10.1111/mec.13636)
Supplement: Supplementary file 4 — Fig. S4 Continuous character map—mean thallus thickness (left) and mean cephalodial volume (right). [file MEC-25-3453-s004.pdf]

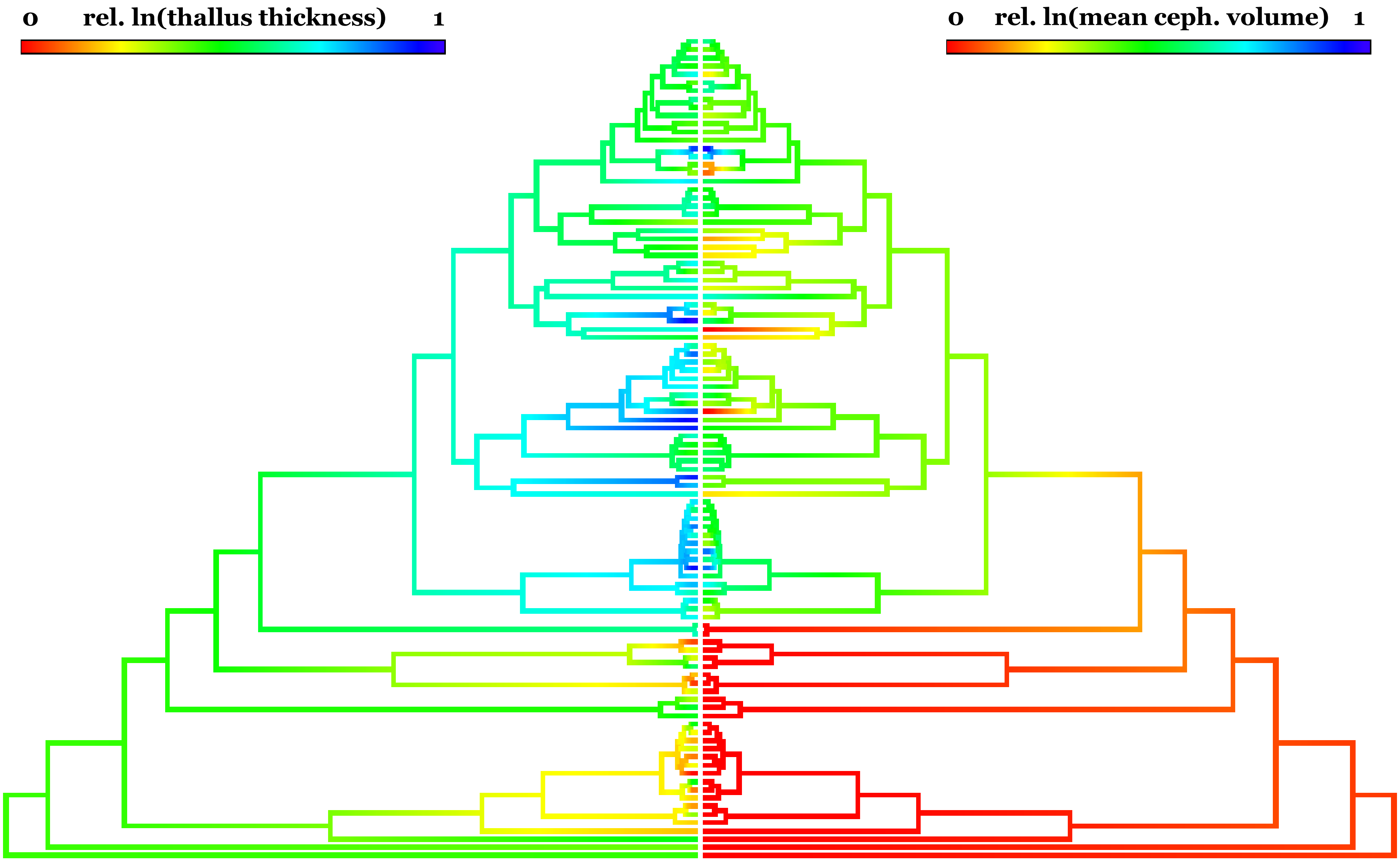

Fig. S4 – Continuous character map – mean thallus thickness (left) and mean cephalodial volume (right).
The natural logarithms of mean thallus thickness and mean cephalodial volume are plotted onto the *BEAST* MCC tree from the run with highest effective sample size (*ESS*). Values are indicated using the colour scales as shown on the top left and top right. The order of nodes is the same as in Fig. 1.
