# Supplementary material for: Escape from the cryptic species trap: lichen evolution on both sides of a cyanobacterial acquisition event
Source: Mol Ecol. 2016 May 11;25(14):3453–68. doi: 10.1111/mec.13636 (PMC5324663; doi:10.1111/mec.13636)
Supplement: Supplementary file 6 — Fig. S6 Distribution of relative mean hymenial volume over beast MCC phylogeny. [file MEC-25-3453-s006.pdf]

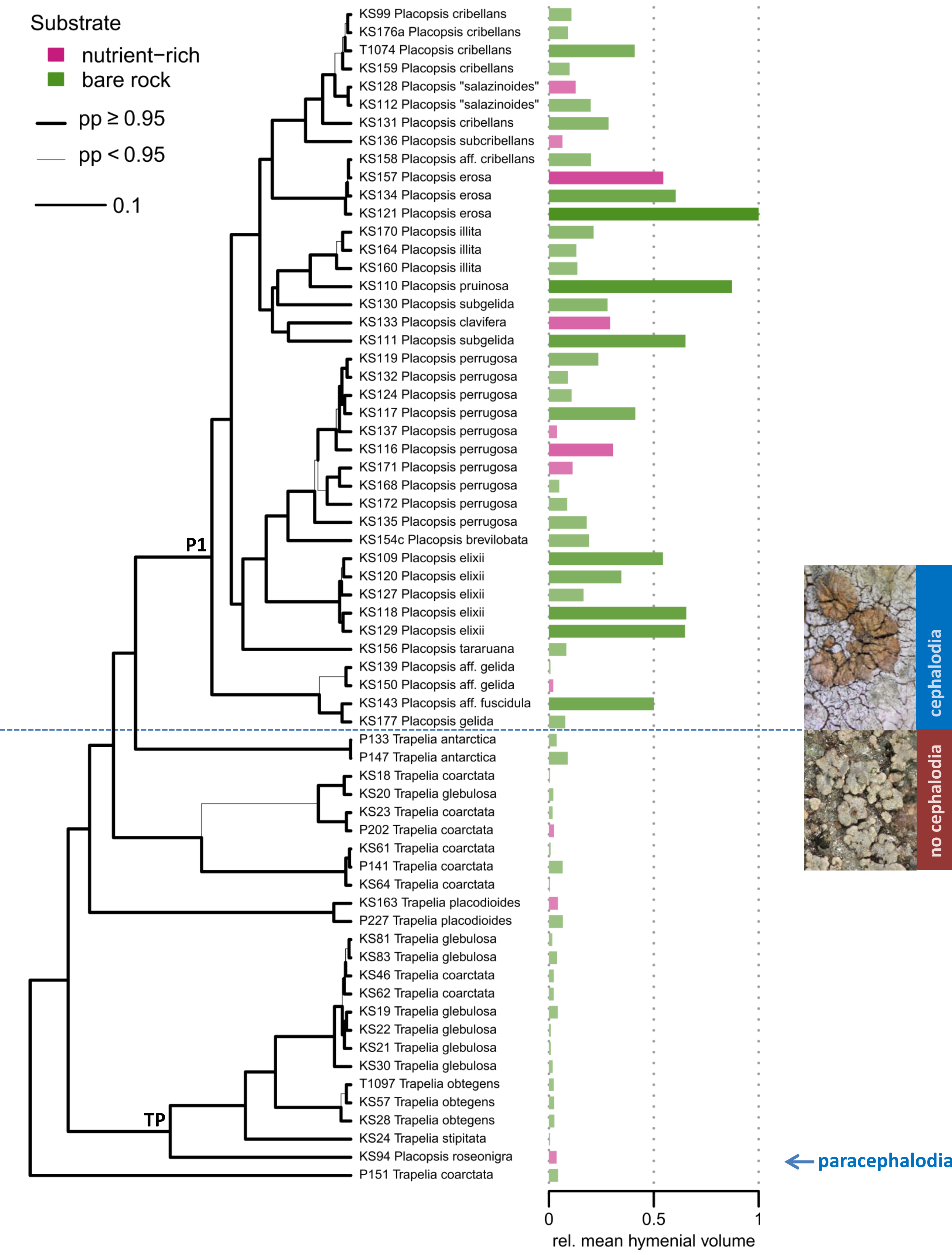

Fig. S6 – Distribution of relative mean hymenial volume over *BEAST* MCC phylogeny. The presence (purple) or absence (green) of potentially nutrient-rich substrate is shown using colour-coding in the bar plot. The transition from *Trapelia* to *Placopsis* sensu stricto is indicated by a dashed blue line. Branch thickness corresponds to posterior probability (*pp*). The scale bar indicates the number of substitutions per nucleotide site. lower picture: *T. glebulosa* (KS47); upper picture: *P. gelida* (KS177); TP: *Trapelia* and *P. roseonigra* clade; P1: *Placopsis* s.str. clade.
