# Supplementary material for: Escape from the cryptic species trap: lichen evolution on both sides of a cyanobacterial acquisition event
Source: Mol Ecol. 2016 May 11;25(14):3453–68. doi: 10.1111/mec.13636 (PMC5324663; doi:10.1111/mec.13636)
Supplement: Supplementary file 8 — Fig. S8 PGLS plot of mean thallus thickness against cephalodial volume for specimens growing on potentially nutrient‐rich substrate. [file MEC-25-3453-s008.pdf]

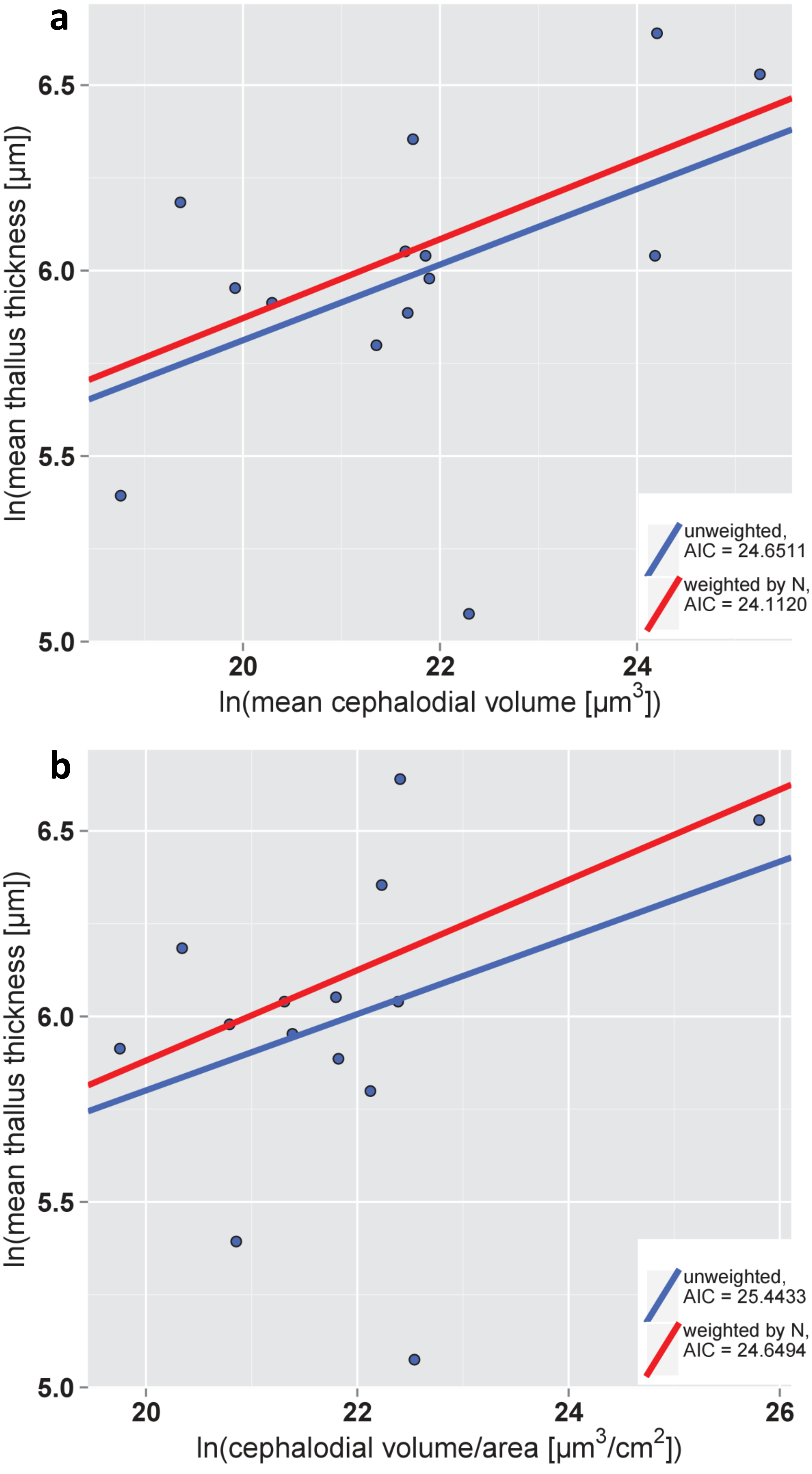

Fig. S8 – PGLS plot of mean thallus thickness against cephalodial volume for specimens growing on potentially nutrient-rich substrate.

a. The natural logarithm of mean thallus thickness is plotted against the natural logarithm of mean cephalodial volume. **b.** The natural logarithm of mean thallus thickness is plotted against the natural logarithm of cephalodial volume per area. blue line: PGLS regression line without weighting by sample size per *bGMYC* cluster; red line: PGLS regression line after weighting by sample size per *bGMYC* cluster.
