# Supplementary material for: Escape from the cryptic species trap: lichen evolution on both sides of a cyanobacterial acquisition event
Source: Mol Ecol. 2016 May 11;25(14):3453–68. doi: 10.1111/mec.13636 (PMC5324663; doi:10.1111/mec.13636)
Supplement: Supplementary file 14 — Table S3 PCR and sequencing primers used in this work. Table S4 Basic descriptive statistics and t‐test results for the Placopsis–Trapelia comparison based on substrate type. Table S5 Results of PGLS analyses with mean thallus thickness as dependent variable. Only specimens growing on potentially nutrient‐rich substrate were included. Table S6 Results of PGLS analyses with mean thallus thickness as dependent variable. Only specimens growing on potentially nutrient‐poor substrate (bare rock surface) were included. Table S7 Results of PGLS analyses with mean hymenial volume as dependent variable. Table S8 Results of PGLS analyses with hymenial volume per area as dependent variable. Table S9 Results of pGLMM analyses with mean hymenial volume as dependent variable. Table S10 Results of pGLMM analyses with hymenial volume per area as dependent variable. [file MEC-25-3453-s014.docx]

**Tab. S3 – PCR and sequencing primers used in this work.**

Sequencing primers are indicated in bold.

| **Primer name** | **Locus** | **Sequence** | **Annealing temp. [°C]** | **Citation** |
| --- | --- | --- | --- | --- |
| ITS1F | ITS1F+5.8S+ITS2 | CTTGGTCATTTAGAGGAAGTAA | 52 | Gardes and Bruns 1993 |
| **ITS4** | ITS1F+5.8S+ITS2 | TCCTCCGCTTATTGATATGC | 52 | White *et al.* 1990 |
| **NS1** | nuSSU | GTAGTCATATGCTTGTCTC | 52 or 54 | White *et al.* 1990 |
| NS17 (nuSSU-0852) | nuSSU | CGTCCCTATTAATCATTACG | 52 or 54 | Gargas and Taylor 1992 |
| **LR0R** | nuLSU | ACCCGCTGAACTTAAGC | 52 | Vilgalys unpublished |
| LR3R | nuLSU | GTCTTGAAACACGGACC | 52 | Hopple and Vilgalys 1999 |
| **LR7** | nuLSU | TACTACCACCAAGATCT | 52 | Vilgalys and Hester 1990 |
| **LRlecF** | nuLSU | CCTCAGTAACGGCGAG | 56 | Schneider *et al.* 2015 |
| **LRlecR** | nuLSU | AGGCTTCGTCACGGAC | 56 | Schneider *et al.* 2015 |
| **mrSSU1** | mtSSU | AGCAGTGAGGAATATTGGTC | 54 | Zoller *et al.* 1999 |
| mrSSU3R | mtSSU | ATGTGGCACGTCTATAGCCC | 54 | Zoller *et al.* 1999 |
| **RPB1-VHAFasc** | RPB1 | ADTGYCCYGGYCATTTYGGT | 52 or 54 | Hofstetter *et al.* 2007 |
| RPB1-Cr | RPB1 | CCNGCDATNTCRTTRTCCATRTA | 52 or 54 | Matheny *et al.* 2002 |
| **fRPB2-5F** | RPB2 | GAYGAYMGWGATCAYTTYGG | 52 | Liu *et al.* 1999 |
| fRPB2-7CR | RPB2 | CCCATRGCTTGYTTRCCCAT | 52 | Liu *et al.* 1999 |
| **MCM7-709for** | MCM7 | ACIMGIGTITCVGAYGTHAARCC | 50 or 52 | Schmitt *et al.* 2009 |
| MCM7-1348rev | MCM7 | GAYTTDGCIACICCIGGRTCWCCCAT | 50 or 52 | Schmitt *et al.* 2009 |
| **EF-983f** | EF1a (1^st^ part) | GCYCCYGGHCAYCGTGAYTTYAT | 56 | Rehner and Buckley 2005 |
| EF-1567R | EF1a (1^st^ part) | ACHGTRCCRATACCACCRATCTT | 56 | Rehner and Buckley 2005 |
| **Efdf** | EF1a (2^nd^ part) | AAGGAYGGNCARACYCGNGARCAYGC | 56 | Rehner unpublished |
| EF-1953-R | EF1a (2^nd^ part) | CCRGCRACRGTRTGTCTCAT | 56 | Rehner unpublished |

**Tab. S4 – Basic descriptive statistics and** $\boldsymbol{t}$**-test results for the *Placopsis*-*Trapelia* comparison based on substrate type.**

The presence (*sub* = 1) or absence (*sub* = 0) of potentially nutrient-rich substrate is indicated. For Welch’s *t*-tests, the natural logarithm of mean thallus thickness was taken.

| **Statistical measure** | ***Placopsis* & *Trapelia*** | ***Placopsis* only** | ***Trapelia* only** |
| --- | --- | --- | --- |
| **Mean (*sub* = 1)** | 367 | 438 | 195 |
| **Median (*sub* = 1)** | 370 | 408 | 215 |
| **Mean (*sub* = 0)** | 283 | 345 | 133 |
| **Median (*sub* = 0)** | 255 | 325 | 112 |
| **Welch’s *t*-test results** | *t* = 2.36; *df* = 31.7;  *p* = 0.0123 | *t* = 2.02; *df* = 21.5;  *p* = 0.0278 | *t* = 2.23; *df* = 6.87;  *p* = 0.0310 |

**Tab. S5 – Results of PGLS analyses with mean thallus thickness as dependent variable.**

Only specimens growing on potentially nutrient-rich substrate were included. Results are shown for regressions weighted by *N*. All morphometric variables were transformed using the natural logarithm prior to analyses. total *df* = 14; residual *df* = 12.

| **Explanatory variable** | **Mean cephalodial volume** | | **Cephalodial volume per area** | |
| --- | --- | --- | --- | --- |
| **Parameter** | **Intercept *a*** | **Slope *b*** | **Intercept *a*** | **Slope *b*** |
| **Value** | 3.74 | 0.11 | 3.45 | 0.12 |
| **Std. error** | 1.17 | 0.05 | 1.87 | 0.09 |
| ***t*-value** | 3.20 | 2.03 | 1.84 | 1.42 |
| ***p*-value** | 0.0076 | 0.0656 | 0.0908 | 0.1812 |

**Tab. S6 – Results of PGLS analyses with mean thallus thickness as dependent variable.**

Only specimens growing on potentially nutrient-poor substrate (bare rock surface) were included. Results are shown for unweighted regressions. All morphometric variables were transformed using the natural logarithm prior to analyses. total *df* = 56; residual *df* = 54.

| **Explanatory variable** | **Mean cephalodial volume** | | **Cephalodial volume per area** | |
| --- | --- | --- | --- | --- |
| **Parameter** | **Intercept *a*** | **Slope *b*** | **Intercept *a*** | **Slope *b*** |
| **Value** | 3.56 | 0.11 | 4.16 | 0.08 |
| **Std. error** | 0.97 | 0.05 | 124 | 0.06 |
| ***t*-value** | 3.68 | 2.39 | 3.34 | 1.36 |
| ***p*-value** | 0.0005 | 0.0203 | 0.0015 | 0.1808 |

**Tab. S7 – Results of PGLS analyses with mean hymenial volume as dependent variable.**

Results are shown for unweighted regressions. All morphometric variables were transformed using the natural logarithm prior to analyses. total *df* = 39; residual *df* = 37.

| **Explanatory variable** | **Mean cephalodial volume** | | **Cephalodial volume per area** | |
| --- | --- | --- | --- | --- |
| **Parameter** | **Intercept *a*** | **Slope *b*** | **Intercept *a*** | **Slope *b*** |
| **Value** | 14.36 | 0.15 | 23.62 | -0.29 |
| **Std. error** | 3.09 | 0.15 | 3.47 | 0.16 |
| ***t*-value** | 4.65 | 1.01 | 6.81 | -1.80 |
| ***p*-value** | 0.0000 | 0.3173 | 0.0000 | 0.0805 |

**Tab. S8 – Results of PGLS analyses with hymenial volume per area as dependent variable.**

Results are shown for unweighted regressions. All morphometric variables were transformed using the natural logarithm prior to analyses. total *df* = 39; residual *df* = 37.

| **Explanatory variable** | **Mean cephalodial volume** | | **Cephalodial volume per area** | |
| --- | --- | --- | --- | --- |
| **Parameter** | **Intercept *a*** | **Slope *b*** | **Intercept *a*** | **Slope *b*** |
| **Value** | 15.19 | 0.21 | 24.25 | -0.22 |
| **Std. error** | 4.24 | 0.20 | 5.17 | 0.25 |
| ***t*-value** | 3.58 | 1.03 | 4.69 | -0.91 |
| ***p*-value** | 0.0010 | 0.3091 | 0.0000 | 0.3706 |

Tab. S9 – Results of pGLMM analyses with mean hymenial volume as dependent variable.

All morphometric variables were transformed using the natural logarithm prior to analyses. *N* = 40.

| **Explanatory variable** | **Mean cephalodial volume** | | **Cephalodial volume per area** | |
| --- | --- | --- | --- | --- |
| **Component** | **Between-species** | **Within-species** | **Between-species** | **Within-species** |
| **Posterior mean (slope *b*)** | -0.17 | 0.37 | -0.52 | -0.18 |
| **Lower 95% credibility interval** | -0.60 | -0.02 | -1.32 | -0.55 |
| **Upper 95% credibility interval** | 0.25 | 0.75 | 0.22 | 0.19 |
| ***p*-value (*pMCMC*)** | 0.4273 | 0.0553 | 0.1743 | 0.3137 |

Tab. S10 – Results of pGLMM analyses with hymenial volume per area as dependent variable.

All morphometric variables were transformed using the natural logarithm prior to analyses. *N* = 40.

| **Explanatory variable** | **Mean cephalodial volume** | | **Cephalodial volume per area** | |
| --- | --- | --- | --- | --- |
| **Component** | **Between-species** | **Within-species** | **Between-species** | **Within-species** |
| **Posterior mean (slope *b*)** | 0.12 | 0.33 | -0.49 | -0.03 |
| **Lower 95% credibility interval** | -0.40 | -0.48 | -1.31 | -0.69 |
| **Upper 95% credibility interval** | 0.69 | 1.03 | 0.36 | 0.64 |
| ***p*-value (*pMCMC*)** | 0.6441 | 0.3794 | 0.2288 | 0.9320 |

**References**

Gardes, M., & Bruns, T. D. (1993) ITS primers with enhanced specificity for basidiomycetes‐application to the identification of mycorrhizae and rusts. *Molecular Ecology*, **2**, 113–118.

Gargas, A., & Taylor, J. W. (1992) Polymerase chain reaction (PCR) primers for amplifying and sequencing nuclear 18S rDNA from lichenized fungi. *Mycologia*, **84**, 589–592.

Hofstetter, V., Miadlikowska, J., Kauff, F., & Lutzoni, F. (2007) Phylogenetic comparison of protein-coding versus ribosomal RNA-coding sequence data: a case study of the Lecanoromycetes (Ascomycota). *Molecular Phylogenetics and Evolution*, **44**, 412–426.

Hopple, J. S., & Vilgalys, R. (1999) Phylogenetic relationships in the mushroom genus *Coprinus* and dark-spored allies based on sequence data from the nuclear gene coding for the large ribosomal subunit RNA: divergent domains, outgroups, and monophyly. *Molecular Phylogenetics and Evolution*, **13**, 1–19.

Liu, Y. J., Whelen, S., & Hall, B. D. (1999) Phylogenetic relationships among ascomycetes: evidence from an RNA polymerse II subunit. *Molecular Biology and Evolution*, **16**, 1799–1808.

Matheny, P. B., Liu, Y. J., Ammirati, J. F., & Hall, B. D. (2002) Using RPB1 sequences to improve phylogenetic inference among mushrooms (*Inocybe*, Agaricales). *American Journal of Botany*, **89**, 688–698.

Rehner, S. A., & Buckley, E. (2005) A *Beauveria* phylogeny inferred from nuclear ITS and EF1-α sequences: evidence for cryptic diversification and links to *Cordyceps* teleomorphs. *Mycologia*, **97**, 84–98.

Schmitt, I., Crespo, A., Divakar, P. K., Fankhauser, J. D., Herman-Sackett, E., Kalb, K., Nelsen, M.P., Nelson, N.A., Rivas-Plata, E., Shimp, A.D., Widhelm, T., & Lumbsch, H. T. (2009) New primers for promising single-copy genes in fungal phylogenetics and systematics. *Persoonia*, **23**, 35–40.

Schneider, K., Resl, P., Westberg, M., & Spribille, T. (2015) A new, highly effective primer pair to exclude algae when amplifying nuclear large ribosomal subunit (LSU) DNA from lichens. *Lichenologist*, **47**, 269–275.

Vilgalys, R., & Hester, M. (1990) Rapid genetic identification and mapping of enzymatically amplified ribosomal DNA from several *Cryptococcus* species. *Journal of Bacteriology*, **172**, 4238–4246.

White, T. J., Bruns, T., Lee, S. J. W. T., & Taylor, J. W. (1990) Amplification and direct sequencing of fungal ribosomal RNA genes for phylogenetics. *PCR protocols: a guide to methods and applications*, **18**, 315–322.

Zoller, S., Scheidegger, C., & Sperisen, C. (1999) PCR primers for the amplification of mitochondrial small subunit ribosomal DNA of lichen-forming Ascomycetes. *Lichenologist*, **31**, 511–516.
